# Electrochemical Performance of ABNO for Oxidation of Secondary Alcohols in Acetonitrile Solution

**DOI:** 10.3390/molecules24010100

**Published:** 2018-12-28

**Authors:** Pengfei Niu, Xin Liu, Zhenlu Shen, Meichao Li

**Affiliations:** 1 College of Chemical Engineering, Zhejiang University of Technology, Hangzhou 310032, China; niu_pengfei@yeah.net (P.N.); lx370682@163.com (X.L.); 2 Research Center of Analysis and Measurement, Zhejiang University of Technology, Hangzhou 310032, China

**Keywords:** electrochemical, ABNO, secondary alcohols, in situ FTIR, ketones

## Abstract

The ketones was successfully prepared from secondary alcohols using 9-azabicyclo[3.3.1]nonane-*N*-oxyl (ABNO) as the catalyst and 2,6-lutidine as the base in acetonitrile solution. The electrochemical activity of ABNO for oxidation of 1-phenylethanol was investigated by cyclic voltammetry, in situ Fourier transform infrared spectroscopy (FTIR) and constant current electrolysis experiments. The resulting cyclic voltammetry indicated that ABNO exhibited much higher electrochemical activity when compared with 2,2,6,6-tetramethylpiperidine-1-oxyl (TEMPO) under the similar conditions. A reasonable reaction mechanism of the electrocatalytic oxidation of 1-phenylethanol to acetophenone was proposed. In addition, a series of secondary alcohols could be converted to the corresponding ketones at room temperature in 80–95% isolated yields.

## 1. Introduction

The carbonyl compounds are important intermediates for synthesis of fine chemicals, such as fragrances, pharmaceuticals, and food additives, and the worldwide demand for carbonyl compounds is increasing every year [1,2,3]. Presently, the preparation of aldehydes and ketones by selective oxidation of the corresponding primary and secondary alcohols is one of the most fundamental functional group transformations in organic chemistry [4,5,6,7]. The stoichiometric or excess amounts of oxidants containing chromium reagents, manganese reagents, or hypervalent iodine are often required among the traditional protocols [8,9,10]. Unfortunately, these oxidants would generate equivalent wastes and pose a risk to the environment [11,12]. It is well known that green chemistry is a crucial principle of chemical research and its application in organic synthesis has attracted more and more attention [13]. From both environmentally benign and sustainable viewpoints, it is a great desire to develop a green and efficient catalyst for the synthesis of the corresponding carbonyl compounds.

Recently, a stable nitroxyl radical 2,2,6,6-tetramethylpiperidine-1-oxyl (TEMPO) has been successfully applied to synthesize aldehydes, nitriles, and imines because of its cleaness and inexpensiveness [14,15,16]. Several combinations of TEMPO and transition metals, especially Fe and Cu, have been widely used for the synthesis of aldehydes from alcohols [17,18,19,20,21,22,23,24,25,26,27,28,29]. Despite of using molecular oxygen as the terminal oxidant, the transition-metal catalyst still exists in these processes. Subsequently, our group developed several catalytic oxidation systems for synthesizing the carbonyl compounds without transition metals, such as TEMPO/HBr/*tert*-butyl nitrite (TBN)/O_2_, TEMPO/TBN/O_2_, TEMPO/2,3-dichloro-5,6-dicyano-1,4-benzoquinone (DDQ)/TBN/O_2_, etc [30,31,32]. Although a lot of meaningful works about the preparation of aldehydes from primary alcohols catalyzed by TEMPO were studied both in homogeneous and heterogeneous conditions, due to the high steric hindrance arising from four methyl group in TEMPO, the synthesis of ketones from secondary alcohols is still a serious challenge [33,34,35,36].

Subsequently, several less hindered nitroxyl radicals, such as 2-azaadamantan-*N*-oxyl (AZADO), 1-Me-AZADO, and 5-F-AZADO, have been utilized by Iwabuchi and co-workers [37,38,39]. Owing to their less steric hindrance around the reaction center, AZADO and its derivatives exhibited high activity at a low loading for the preparation of carbonyl compounds from selective oxidation of high hindered alcohols as compared with TEMPO [40]. However, AZADOs should be synthesized through long and complicated processes. Another nitroxyl radical, 9-azabicyclo[3.3.1]nonane-*N*-oxyl (ABNO) has the similar activity with AZADOs and it could be prepared via only four steps [41]. It put forward the possibility to prepare the corresponding carbonyl compounds from primary and secondary alcohols with ABNO as the catalyst under mild conditions. Previously, the ABNO/TBN/O_2_ system for the aerobic oxidation of secondary alcohols in water has been developed [42]. This reaction should be performed at 80 °C under 0.3–0.5 Mpa of oxygen.

**Scheme 1:**
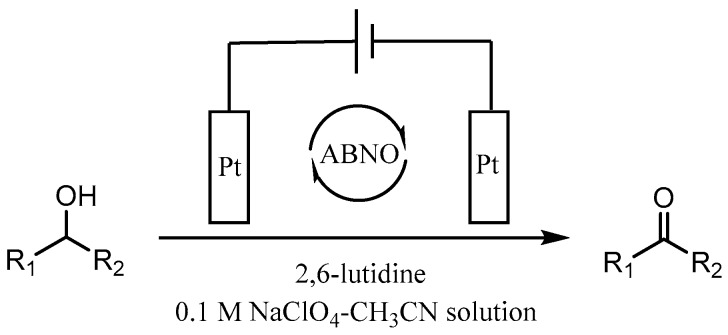
The electrochemical synthesis of ketones from secondary alcohols.

As we all know, the electrochemical method in synthesis possesses the advantages of friendly environment, low cost, and high atom utilization, paving a new way for production of many fine chemicals [43,44,45,46]. In 2012, Raja et al. disclosed an electrochemical method to achieve the preparation of aromatic aldehydes with sodium nitrate as an effective redox mediator in biphasic medium [47]. However, the primary alcohols substituted with strong electron-withdrawing group, such as -nitro and some of secondary alcohols gave low product yields. Lately, we prepared a series of TEMPO-modified polymer electrodes, which could be used successfully for the selective oxidation of alcohols to aldehydes [48,49,50]. It is well known that the detailed information of the electrochemical behaviour of organic compounds is crucial for electrochemical synthesis. Stahl’s group reported that 4-acetamido-TEMPO (ACT) possessed of the superior activity than AZADO and ABNO at high pH with electrocatalytic studies about the nitroxyl/oxoammonium redox potential [51]. In continuation with our previous work about the synthesis of aldehydes from alcohols, we try to study the electrochemical performance of ABNO as the catalyst for the selective oxidation of secondary alcohols in the presence of 2,6-lutidine in acetonitrile solution. In this work, the electrochemical behaviour of ABNO for the oxidation of 1-phenylethanol to acetophenone on the Pt electrode was studied to a better understanding of the catalytic properties (Scheme 1).

## 2. Results and Discussion

### 2.1. Cyclic Voltammetric Study for Oxidation of 1-Phenylethanol

**Figure 1:**
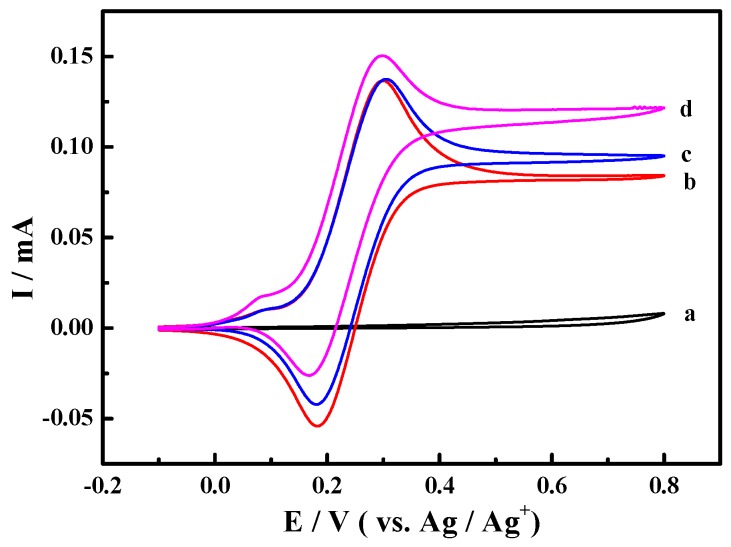
Cyclic voltammograms of Pt electrode in 0.1 M NaClO_4_-CH_3_CN solution with (**a**) 1-phenylethanol (1.0 mmol) and 2,6-lutidine (1.0 mmol); (**b**) active oxoammonium cations (ABNO) (0.1 mmol); (**c**) ABNO (0.1 mmol) and 1-phenylethanol (1.0 mmol); and, (**d**) ABNO (0.1 mmol), 1-phenylethanol (1.0 mmol) and 2,6-lutidine (1.0 mmol) at the scan rate of 50 mV·s^−1^.

The cyclic voltammograms for the oxidation of 1-phenylethanol in 0.1 M NaClO_4_-CH_3_CN solution using ABNO as the catalyst were shown in Figure 1. We first started out investigation in 0.1 M NaClO_4_-CH_3_CN solution containing 1-phenylethanol (1.0 mmol) and 2,6-lutidine (1.0 mmol) at Pt electrode (Figure 1a). No obvious reaction peaks appeared in the range of scanning potential between −0.1 and 0.8 V. As shown in Figure 1b, a pair of reversible redox peaks was well centered at about 0.25 V in the presence of ABNO (0.1 mmol). The oxidation peak at 0.3 V corresponded to the one-electron oxidation of nitroxyl radical to oxoammonium ion (ABNO^+^) rather than the response of 1-phenylethanol or 2,6-lutidine [51]. By the addition of 1-phenylethanol, the reaction was so weak that we could not detect the obvious change about the oxidation peak current (Figure 1c). However, when the base 2,6-lutidine was added to the reaction solution, the apparent increase of oxidation peak current from 1.37 mA to 1.51 mA was observed (Figure 1d). It certified that the active oxoammonium cations reacted with 1-phenylethanol on the surface of the working electrode and 2,6-lutidine was beneficial to facilitate the oxidation of 1-phenylethanol [52].

**Figure 2:**
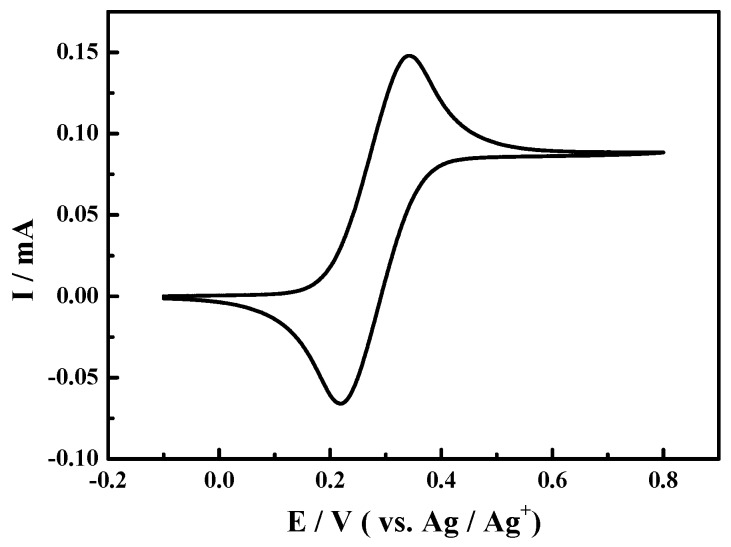
Cyclic voltammogram of Pt electrode in 0.1 M NaClO_4_-CH_3_CN solution with 2,2,6,6-tetramethylpiperidine-1-oxyl (TEMPO) (0.1 mmol) at the scan rate of 50 mV·s^−1^.

For comparison, other experiments for oxidation of 1-phenylethanol in 0.1 M NaClO_4_-CH_3_CN solution using TEMPO as the catalyst under the similar conditions have been conducted. As shown in Figure 2, the redox peaks at about 0.34 and 0.22 V could be seen in the presence of TEMPO (0.1 mmol), which were corresponded to one electron transfer from TEMPO to TEMPO^+^ [53]. When 1-phenylethanol and 2,6-lutidine were added to the above reaction solution sequentially, the corresponding cyclic voltammograms were almost overlapped and were omitted in Figure 2 hence.

**Figure 3:**
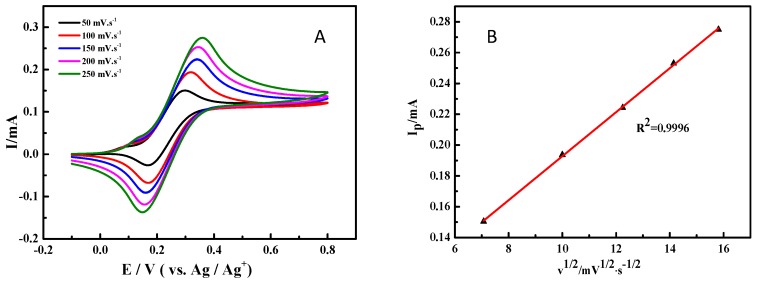
(**A**) Cyclic voltammograms for the oxidation of 1-phenylethanol (1.0 mmol) with ABNO (0.1 mmol) and 2,6-lutidine (1.0 mmol) in 0.1 M NaClO_4_-CH_3_CN solution at various scan rates. (**B**) Linear plot between the oxidation peak current (I_p_) and the square root of scan rate.

To futher investigate the electrochemical behavior of ABNO for 1-phenylethanol oxidation, the cyclic voltammograms with various scan rates were studied and shown in Figure 3A. Due to the high solution resistance in CH_3_CN solution, the peak-to-peak separation between the oxidation and reduction peaks was about 100 mV at the scan rate of 50 mV·s^−1^, which was slightly more than the 59 mV separation [54]. Nevertheless, with the increasing of scanning rate, there was a less shift in the oxidation peak, suggesting that the electrochemical reaction of ABNO still had good reversibility [55]. The excellent linear correlation between the oxidation peak current (I_p_) and the square root of scan rate (Figure 3B) exhibited that the electrode reaction was also a diffusion-controlled process, which provided further evidence for reacting on the surface of working electrode [56].

### 2.2. In situ FTIR Spectroscopic Analysis

**Figure 4:**
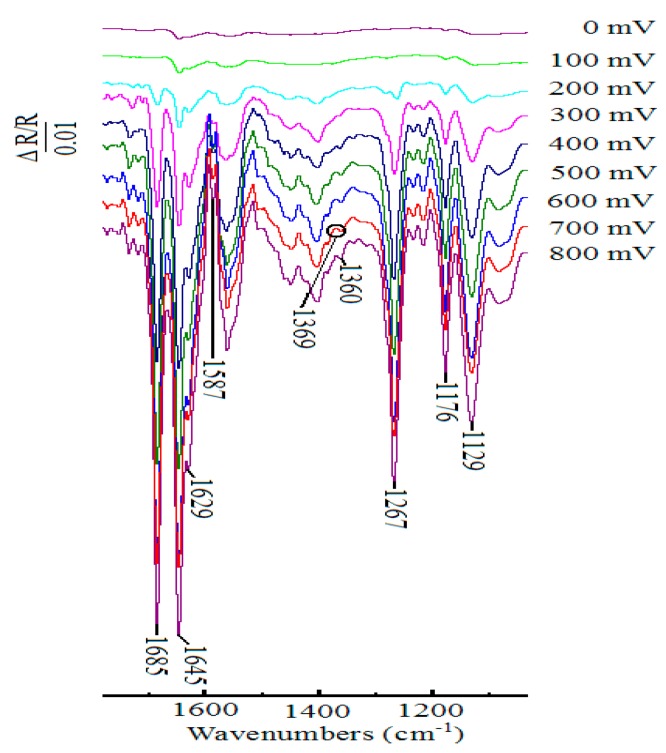
In situ FTIR spectra collected on Pt disk electrode during the oxidation of 1-phenylethanol (1.0 mmol) in the presence of ABNO (0.1 mmol) and 2,6-lutidine (1.0 mmol) in 0.1 M NaClO_4_-CH_3_CN solution in a short time interval 10 s.

In situ FTIR spectroscopy is one of the most useful techniques in electrochemistry today, and it can be used to study the organic reaction at the electrode surface. Figure 4 showed the in situ FTIR spectra that were obtained during the oxidation of 1-phenylethanol in the presence of ABNO and 2,6-lutidine in 0.1 M NaClO_4_-CH_3_CN solution at the scanning potentials varying from 0 to 800 mV. When the sample potential reached to 200 mV, some weak bands emerged gradually. With the increasing of potential, the corresponding infrared spectra signals could be seen clearly at approximately 400 mV. The downward bands at 1646, 1629, and 1587 cm^−1^ were contributed to ring stretching vibration of 2,6-lutidinium cation [57]. The band at 1176 cm^−1^ was related to C-H in-plane bending of 2,6-lutidinium cation [58,59]. It confirmed that 2,6-lutidine got a hydrogen proton to become 2,6-lutidinium cation during the electrocatalytic process [60,61]. Meanwhile, three negative-going bands at 1685, 1360, and 1267 cm^−1^ were detected, which were attributed to C=O stretching vibration, C-H deformation vibration in the methyl group, and skeleton vibration of acetophenone, respectively [62]. Moreover, a weak but important upward band of N-O stretching vibration, showing the participation of ABNO in this reaction, was observed at 1369 cm^−1^ [49]. The other band located at 1129 cm^−1^ was assigned to ClO_4_^−^ ions of supporting electrolyte [63,64]. However, with the interference of the nearby signal peaks at about 1280 and 1627 cm^−1^, the ring stretching modes of 2,6-lutidinium cation and N^+^=O stretching vibration of oxoammonium ion could not be obviously found [48]. From the above results, we could get the conclusion that 1-phenylethanol was oxidized to acetophenone with ABNO as the catalyst and 2,6-lutidine as the base.

**Figure 5:**
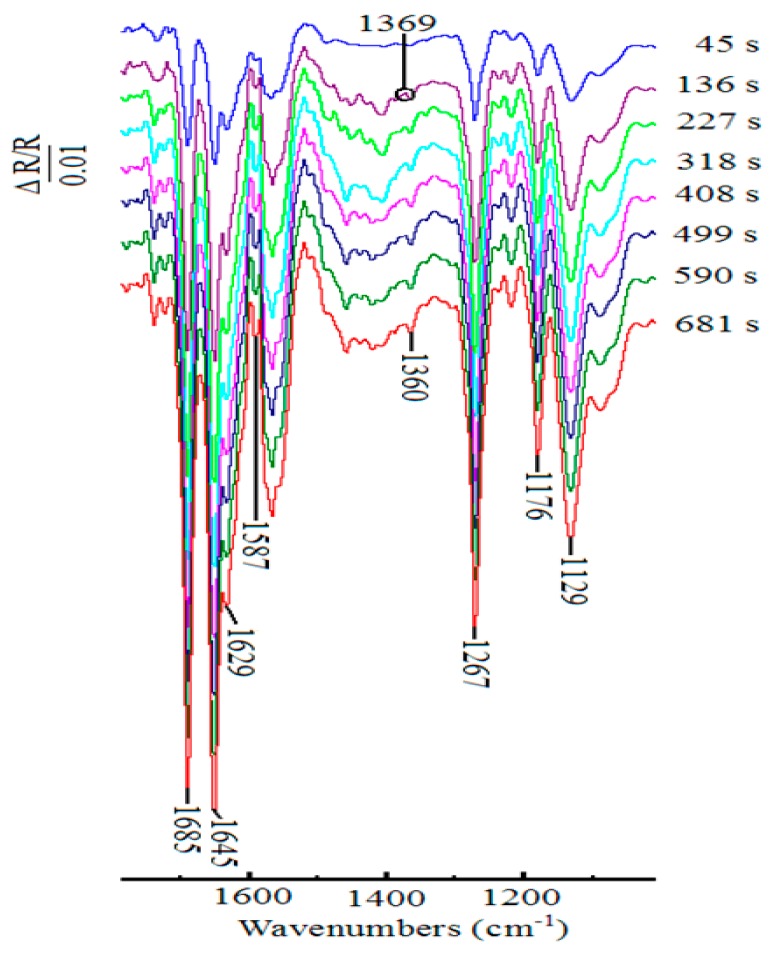
In situ time-resolved FTIR spectra collected on Pt disk electrode during the oxidation of 1-phenylethanol (1.0 mmol) in the presence of ABNO (0.1 mmol) and 2,6-lutidine (1.0 mmol) in 0.1 M NaClO_4_-CH_3_CN solution at 400 mV.

To further observe the spectral changes with increasing time, the in situ time-resolved FTIR spectra experiment was carried out during the oxidation of 1-phenylethanol on Pt disk electrode with ABNO as the catalyst at 400 mV. The resulting spectra were displayed in Figure 5. The seven negative signals at 1685, 1360, and 1267 cm^−1^ and at 1645, 1629, 1587, and 1176 cm^−1^, illustrated to acetophenone and 2,6-lutidinium cation, respectively, could still be seen clearly [58,59,62]. The intensity of these bands significantly increased with the increasing of time. Meanwhile, no new bands emerged, which showed that no new reaction occurred on the electrode surface.

**Figure 6:**
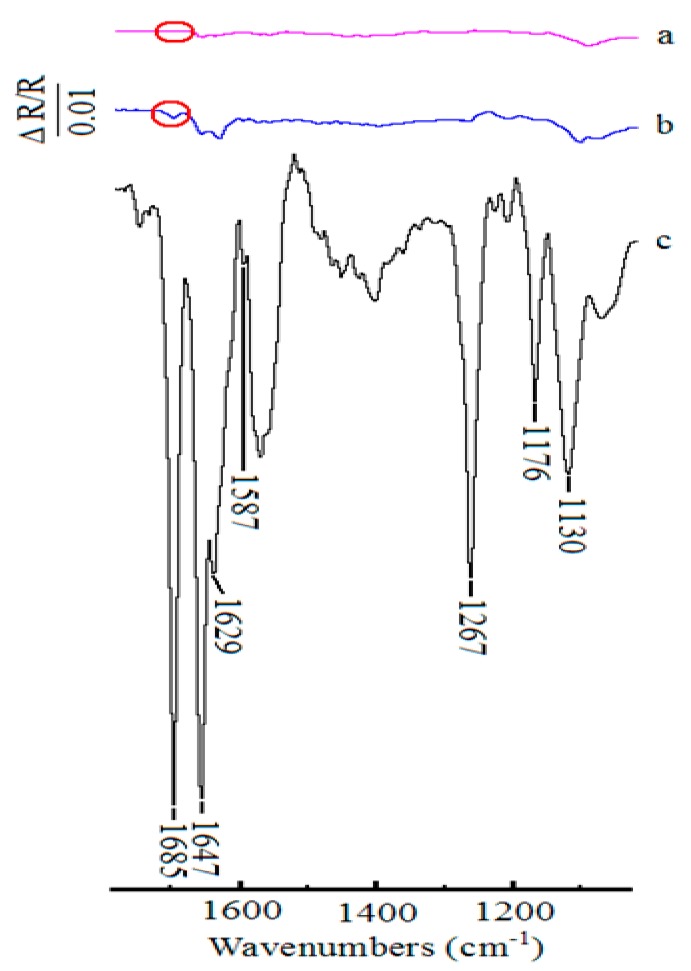
Comparison of in situ FTIR spectra collected on Pt disk electrode during the oxidation of 1-phenylethanol (1.0 mmol) in the presence of 2,6-lutidine (1.0 mmol) at 400 mV. (**a**) without any catalyst, (**b**) with TEMPO (0.1 mmol) as the catalyst, (**c**) with ABNO (0.1 mmol) as the catalyst.

For comparision, the in situ FTIR spectra collected during the oxidation of 1-phenylethanol in the presence of 2,6-lutidine with TEMPO as the catalyst and without any catalyst in 0.1 M NaClO_4_-CH_3_CN solution under the similar conditions were obtained. The spectra at 400 mV were put together and presented in Figure 6. As shown in Figure 6a, no additional bands were observed except for the band of supporting electrolyte at Pt electrode without any catalyst. In addition, with the addition of TEMPO as the catalyst in the reaction solution, all of the characteristic bands changed weakly (Figure 6b). Only with ABNO as the catalyst in the reaction solution, the intensity of the bands enhanced markedly, which was identify with cyclic voltammograms results (Figure 6c). Thus, ABNO could act as an efficient catalyst for the oxidation of secondary alcohols instead of TEMPO under the same conditions [65,66].

**Scheme 2:**
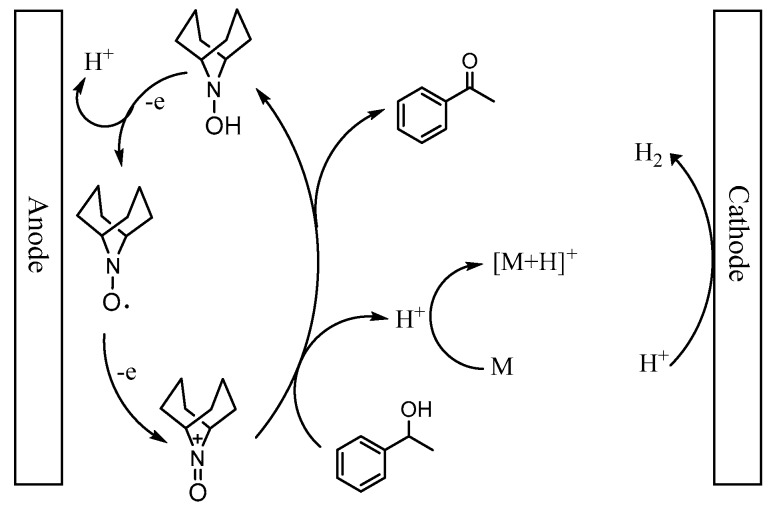
A plausible mechanism for the oxidation of 1-phenylethanol in the presence of ABNO and 2,6-lutidine (M).

Based on the above observation and relevant literatures, a plausible mechanism for the oxidation of 1-phenylethanol was presented in Scheme 2. The catalyst ABNO could be oxidized to generate the active oxoammonium cations (ABNO^+^) via one electronic transfer process on the anode electrode [36,67]. Subsequently, 1-phenylethanol reacted with ABNO^+^ to give acetophenone and 9-hydroxy-9-azabicyclo[3.3.1]nonane (ABNOH), accompanied by one hydrogen proton generation [42,68]. Meanwhile, ABNOH was regenerated to ABNO [51,69]. The base 2,6-lutidine got the hydrogen proton to form 2,6-lutidinium cation during the oxidation reaction [48]. In addition, H_2_ was generated as the side product on the cathode electrode [54].

### 2.3. Preparation Electrolysis

Inspired by these results, the scope of this electrochemical method for the oxidation of secondary alcohols was explored. As shown in Table 1, 1-phenylethanol could be converted completely to acetophenone in 98% GC internal standard yield (entry 1). Due to the volatility of acetophenone during the process of purification and concentration, the isolated yield was relatively low (80%). Meanwhile, we tried to calculate the faradaic efficiency in this electrochemical process. The faradaic efficiencies with 1-phenylethanol were about 81.4% at 6 h, with 92% conversion and 99% selectivity. With the reaction proceeding, the concentration of the substrate was decreased and the reaction slowed down gradually. The faradaic efficiency was about 61.8% at 8.5 h. Similarly, a series of secondary benzyl alcohols with both electro-donating and electron-withdrawing substituents in phenyl group were provided in good to excellent isolated yields (entires 2–11). For example, four secondary benzylic alcohols with electro-donating group were converted into the corresponding ketones with good isolated yields under the standard conditions (entries 5–8). Meanwhile, halide substituents products such as chloro-acetophenone (*o*-, *m*-, and *p*-), 4-bromo-acetophenone and 4-fluoro-acetophenone were all obtained in excellent isolated yields (entries 2–4,10,11). Furthermore, 1-(4-nitrophenyl)ethanol could afford the target product efficiently (entry 9). When 1-phenylpropan-1-ol was chosen as the substrate, a full conversion with 88% isolated yield could be achieved (entry 12). Other excellent results were also obtained, including 1-phenyl-1-butanol and 1-(*p*-tolyl)propan-1-ol (entries 13 and 14). Notably, the conversion of 2-methyl-1-phenylpropan-1-ol could reach to 98% by prolonging the reaction time to 11 h (entry 15). The selectivity to 2-methylpropiophenone was 92% and the main by-product was benzaldehyde. Next, the polycyclic and heterocyclic substrates were smoothly converted to the corresponding ketones with satisfying isolated yields (entries 16–18). In order to explore the applicability of the protocol for unactivated secondary aliphatic alcohols, cycloheptanol and 2-octanol were tested. Fortunately, the high GC yields of their corresponding ketones were received in 98% and 90%, respectively (entries 19 and 20).

## 3. Materials and Methods

### 3.1. Catalyst Preparation and Reagents

ABNO was synthesized in our laboratory according to the available procedures [41]. Other chemicals and solvents were purchased from the supplier and were used as received. In this work, the racemic secondary alcohols were used.

### 3.2. Cyclic Voltammetry Study

The cyclic voltammetric study was carried out by using CHI620B electrochemical workstation (CH Instrument Inc., Austin, TX, USA) with an “L” type Pt electrode as the working electrode. A big square platinum sheet (1.5 cm in length) and a Ag/Ag^+^ electrode (0.1 M AgNO_3_ in acetonitrile) were employed as the counter electrode and the reference one, respectively. The experiments were performed in 0.1 M NaClO_4_-CH_3_CN solution at the scan rate of 50 mV·s^−1^ at room temperature. All of the potentials in this article were referred to the Ag/Ag^+^ electrode (0.1 M AgNO_3_ in acetonitrile).

### 3.3. In Situ FTIR Spectroscopic Study

The in situ infrared spectroscopy experiments were performed on 263 A Potentiostat/Galvanostat and Nicolet 670 FTIR spectrometer (Thermo Fisher Nicolet, Waltham, MA, USA), equipped with a refrigerated MCT-A detector and KBr beam splitter. The disk electrode of Pt (0.6 cm in diameter) was used as the working electrode. A three-electrode spectro-electrochemical cell equipped with CaF_2_ window as the IR window was used for collecting the interferograms. Each single-beam spectrum was collected and two hundred interferograms were coadded at a spectral resolution of 8 cm^−1^. The sample potentials varied in a stepwise fashion from 0 to 800 mV and the reference potential was fixed at −100 mV [70,71,72].

### 3.4. Preparation Electrolysis Experiments

The preparative electrolysis experiments were conducted with in an undivided cell containing 0.1 M NaClO_4_-CH_3_CN solution (15 mL), alcohol substrate (1.0 mmol), ABNO (0.1 mmol), and 2,6-lutidine (1.0 mmol) at a constant current of 10.0 mA with moderate magnetic stirring for 8.5 h in the atmosphere. Two square platinum sheets were employed as the anode and cathode, respectively. The electrolytic reaction was monitored by gas chromatography (GC) on a GC-2010 system (Shimadzu, Kyoto, Japan) equipped with a SH-Rtx-Was polar column and a flame ionization detector (FID). Both the injector and detector were maintained at 220 °C, the carrier gas is nitrogen, and the flow rate is 1.2 mL/min. The initial oven temperature of 100 °C was held for 2 min and then ramped up at 15 °C per min to 220 °C. This final temperature was held for 8 min. After the reaction was finished, the resulting mixture was concentrated in a rotary evaporator (Heidolph, Schwabach, Germany) and purified by column chromatography on silica gel using petroleum and ethyl acetate 15:1) as eluent to afford the products. The products were confirmed by GC-MS, ^1^H-NMR, and ^13^C-NMR. NMR spectroscopy was carried out on a Bruker Avance III spectrometer (Bruker, Fällanden, Switzerland). The GC-MS analysis was measured on Thermo Trace ISQ instrument (Thermo Fisher Nicolet, Waltham, MA, USA) with TG 5MS capillary column.

*Acetophenone* (colorless oil, yield 80%): ^1^H-NMR (500 MHz, CDCl_3_) δ 7.94–7.92 (m, 2H), 7.55–7.51 (m,1H), 7.44–7.41 (m, 2H), 2.57 (s, 3H). ^13^C-NMR (125 MHz, CDCl_3_) δ 198.0, 136.9, 132.9, 128.4, 128.1, 26.4. GC-MS (EI): *m*/*z*: 120.14 [M^+^].

*1-(4-Chlorophenyl)ethanone* (colorless oil, yield 94%): ^1^H-NMR (500 MHz, CDCl_3_) δ 7.89–7.87 (m, 2H), 7.42–7.41 (m, 2H), 2.57 (s, 3H). ^13^C-NMR (125 MHz, CDCl_3_) δ 196.7, 139.5, 135.4, 129.7, 128.8, 26.5. GC-MS (EI): *m*/*z*: 154.03 [M^+^].

*1-(3-Chlorophenyl)ethanone* (colorless oil, yield 88%): ^1^H-NMR (500 MHz, CDCl_3_) δ 7.92–7.91 (m, 1H), 7.83–7.81 (m, 1H), 7.54–7.51 (m, 1H), 7.42–7.39 (m, 1H), 2.59 (s, 3H). ^13^C-NMR (125 MHz, CDCl_3_) δ 196.6, 138.6, 134.9, 133.0, 129.9, 128.3, 126.4, 26.6. GC-MS (EI): *m*/*z*: 154.17 [M^+^].

*1-(2-Chlorophenyl)ethanone* (colorless oil, yield 87%): ^1^H-NMR (500 MHz, CDCl_3_) δ 7.56–7.54 (m, 1H), 7.43–7.37 (m, 2H), 7.34–7.31 (m, 1H), 2.65 (s, 3H). ^13^C-NMR (125 MHz, CDCl_3_) δ 200.4, 139.1, 132.0, 131.3, 130.6, 129.4, 126.9, 30.7. GC-MS (EI): *m*/*z*: 154.11 [M^+^].

*1-(4-Fluorophenyl)ethanone* (colorless oil, yield 85%): ^1^H-NMR (500 MHz, CDCl_3_) δ 8.00–7.96 (m, 2H), 7.15–7.10 (m, 2H), 2.58 (s, 3H). ^13^C-NMR (125 MHz, CDCl_3_) δ 196.4, 165.8 (d, *J* = 254.8 Hz), 133.6 (d, *J* = 2.4 Hz), 130.9 (d, *J* = 9.3 Hz), 115.6 (d, *J* = 21.9 Hz), 26.5. GC-MS (EI): *m*/*z*: 138.06 [M^+^].

*1-(4-Bromophenyl)ethanone* (white solid, yield 92%): ^1^H-NMR (500 MHz, CDCl_3_) δ 7.83–7.80 (m, 2H), 7.61–7.59 (m, 2H), 2.58 (s, 3H). ^13^C-NMR (125 MHz, CDCl_3_) δ 196.9, 135.8, 131.9, 129.8, 128.3, 26.5. GC-MS (EI): *m*/*z*: 197.98, 200.04 [M^+^].

*1-(4-Methylphenyl)ethanone* (colorless oil, yield 92%): ^1^H-NMR (500 MHz, CDCl3) δ 7.86–7.84(m, 2H), 7.28–7.24 (m, 2H), 2.56 (s, 3H), 2.40 (s, 3H). ^13^C-NMR (125 MHz, CDCl_3_) δ 197.7, 143.8, 134.7, 129.2, 128.4, 26.4, 21.5. GC-MS (EI): *m*/*z*: 134.15 [M^+^].

*1-(3-Methylphenyl)ethanone* (colorless oil, yield 93%): ^1^H-NMR (500 MHz, CDCl_3_) δ 7.77–7.74(m, 2H), 7.38–7.33 (m, 2H), 2.59 (s, 3H), 2.41 (s, 3H). ^13^C-NMR (125 MHz, CDCl_3_) δ 198.3, 138.3, 137.1, 133.8, 128.7, 128.4, 125.5, 26.6, 21.2. GC-MS (EI): *m*/*z*: 134.10 [M^+^].

*1-(2-Methylphenyl)ethanone* (colorless oil, yield 95%): ^1^H-NMR (500 MHz, CDCl_3_) δ 7.71–7.69(m, 1H), 7.40–7.36 (m, 1H), 7.28–7.24 (m, 2H), 2.58 (s, 3H), 2.54 (s, 3H). ^13^C-NMR (125 MHz, CDCl_3_) δ 201.7, 138.3, 137.6, 132.0, 131.5, 129.3, 125.6, 29.5, 21.5. GC-MS (EI): *m*/*z*: 134.09 [M^+^].

*1-(4-Methoxyphenyl)ethanone* (colorless oil, yield 89%): ^1^H-NMR (500 MHz, CDCl_3_) δ 7.94–7.91 (m, 2H), 6.94–6.91 (m, 2H), 3.86 (s, 3H), 2.55 (s, 3H). ^13^C-NMR (125 MHz, CDCl_3_) δ 196.7, 163.5, 130.5, 130.3, 113.6, 55.4, 26.3. GC-MS (EI): *m*/*z*: 150.14 [M^+^].

*1-(4-Nitrophenyl)ethanone* (yellow crystal powder, yield 90%): ^1^H-NMR (500 MHz, CDCl_3_) δ 8.28–8.27 (m, 2H), δ 8.11–80.08 (m, 2H), δ 2.66 (s, 3H); _13_C-NMR (125 MHz, CDCl_3_) δ 196.3, 150.3, 141.3, 129.2, 123.7, 26.9. GC-MS (EI): *m*/*z*: 165.05 [M^+^].

*Propiophenone* (colorless oil, yield 88%): ^1^H-NMR (500 MHz, CDCl_3_) δ 7.99–7.97 (m, 2H), 7.58–7.55(m, 1H), 7.49–7.45 (m, 2H), 3.04–3.00 (m, 2H), 1.24 (t, *J* = 7.2 Hz, 3H). ^13^C-NMR (125 MHz, CDCl_3_) δ 200.9, 137.0, 132.9, 128.6, 128.0, 31.8, 8.3. GC-MS (EI): *m*/*z*: 134.16 [M^+^].

*Phenylbutanone* (colorless oil, yield 92%): ^1^H-NMR (500 MHz, CDCl_3_) δ 7.95–7.93 (m, 2H), 7.54–7.50(m, 1H), 7.44–7.41 (m, 2H), 2.94–2.91 (m, 2H), 1.79–1.72 (m, 2H), 0.99 (t, *J* = 7.4 Hz, 3H). ^13^C-NMR (125 MHz, CDCl_3_) δ 200.2, 137.0, 132.7, 128.4, 127.9, 40.3, 17.6, 13.7. GC-MS (EI): *m*/*z*: 148.17 [M^+^].

*2-Methylpropiophenone* (colorless oil, yield 89%): ^1^H-NMR (500 MHz, CDCl_3_) δ 7.98–7.96 (m, 2H), 7.58–7.54 (m, 1H), 7.49–7.46 (m, 2H), 3.60–3.54 (m, 1H), 1.23 (d, *J* = 6.9 Hz, 6H). ^13^C-NMR (125 MHz, CDCl_3_) δ 204.5, 136.3, 132.8, 128.6, 128.3, 35.3, 19.1. GC-MS (EI): *m*/*z*: 148.18 [M^+^].

*1-(4-Methylphenyl)propan-2-one* (colorless oil, yield 93%): ^1^H-NMR (500 MHz, CDCl_3_) δ 7.87 (d, *J* = 8.2 Hz, 2H), 7.28–7.25 (m, 2H), 3.00–2.96 (m, 2H), 2.41 (s, 3H), 1.22 (t, *J* = 7.3 Hz, 3H). ^13^C-NMR (125 MHz, CDCl_3_) δ 200.5, 143.5, 134.4, 129.2, 128.1, 31.6, 21.5, 8.3. GC-MS (EI): *m*/*z*: 148.16 [M^+^].

*1-Tetralone* (white solid, yield 90%): ^1^H-NMR (500 MHz, CDCl_3_) δ 8.04–8.03 (m, 1H), 7.48–7.45 (m, 1H), 7.30 (t, *J* = 7.5 Hz, 1H), 7.25 (d, *J* = 7.6 Hz, 1H), 2.97 (t, *J* = 6.1 Hz, 2H), 2.67–2.64 (m, 2H), 2.17–2.12 (m, 2H). ^13^C-NMR (125 MHz, CDCl_3_) δ 198.3, 144.4, 133.3, 132.6, 128.7, 127.1, 126.6, 39.1, 29.7, 23.2. GC-MS (EI): *m*/*z*: 146.17 [M^+^].

*Benzophenone* (white solid, yield 95%): ^1^H-NMR (500 MHz, CDCl_3_) δ 7.82 (d, *J* = 7.4 Hz, 4H), 7.60 (t, *J* = 7.4 Hz, 2H), 7.49 (t, *J* = 7.6 Hz, 4H). ^13^C-NMR (125 MHz, CDCl_3_) δ 196.7, 137.6, 132.4, 130.0, 128.2. GC-MS (EI): *m*/*z*: 182.00 [M^+^].

*1-(Thiophen-2-yl)ethanone* (yellow oil, yield 80%): ^1^H-NMR (500 MHz, CDCl_3_) δ 7.69–7.68 (m, 1H), 7.63–7.62 (m, 1H), 7.12–7.10 (m, 1H), 2.55 (s, 3H). ^13^C-NMR (125 MHz, CDCl_3_) δ 190.6, 144.5, 133.7, 132.4, 128.0, 26.8. GC-MS (EI): *m*/*z*: 125.97 [M^+^].

*Cycloheptanone* (colorless oil, yield 57%): ^1^H-NMR (500 MHz, CDCl_3_) δ 2.49–2.47 (m, 4H), 1.70–1.64 (m, 8H); ^13^C-NMR (125 MHz, CDCl_3_) δ 215.3, 43.8, 30.4, 24.3. GC-MS (EI): *m*/*z*: 112.11 [M^+^].

## 4. Conclusions

In conclusion, the electrochemical behaviour of the nitroxyl radical ABNO for the oxidation of 1-phenylethanol in acetonitrile solution have been studied. The electrochemical measurements revealed that ABNO showed reversible redox behavior and it had lower potential than TEMPO. The oxoammonium ion (ABNO^+^) was generated by single-electron oxidation. According to the in situ FTIR spectra results, the base 2,6-lutidine received a hydrogen proton to become 2,6-lutidinium cation, which was a crucial process during the synthesis of acetophenone from 1-phenylethanol. Under mild reaction conditions, a variety of substrates, including aromatic, heteroaromatic, and aliphatic secondary alcohols could be converted to the corresponding ketones with good to excellent isolated yields.

## Figures and Tables

**Table 1:** Electrochemical conversion of various secondary alcohols to ketones ^a^.

Entry	Substrate	Product	Conversion ^b^ (%)	Selectivity ^b^ (%)	Yield ^c^ (%)
1	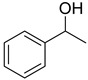	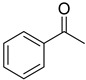	>99	>99	80 (98)
2	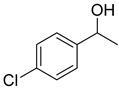	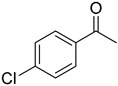	>99	98	94
3	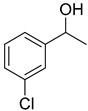	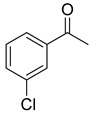	>99	97	88
4	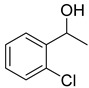	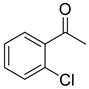	>99	96	87
5	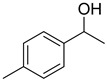	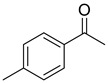	>99	97	92
6	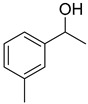	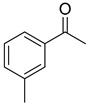	>99	97	93
7	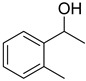	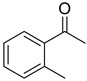	>99	98	95
8	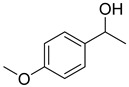	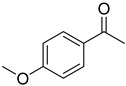	>99	97	89
9	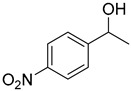	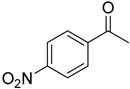	>99	96	90
10	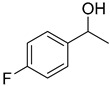	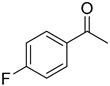	>99	97	85
11	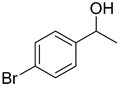	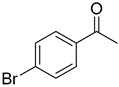	>99	97	92
12	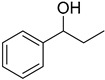	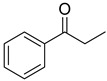	99	99	88
13	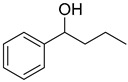	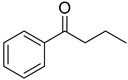	98	98	92
14	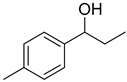	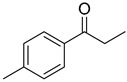	>99	97	93
15 ^d^	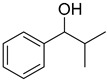	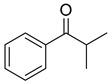	98	92	89
16	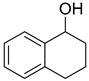	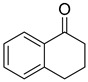	>99	99	90
17	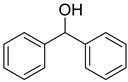	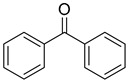	>99	99	95
18	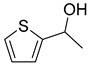	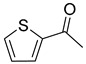	>99	99	80
19	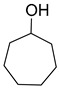	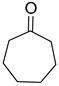	99	99	98 ^e^
20	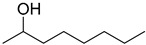	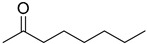	92	98	90 ^e^

^a^ Electrolytic conditions: substrate (1.0 mmol), ABNO (0.1 mmol), 2,6-lutidine (1.0 mmol), 0.1 M NaClO_4_-CH_3_CN solution (15 mL), undivided cell, constant current 10 mA, electrolysis time 8.5 h, room temperature. ^b^ Determined by GC with peak area normalization method. ^c^ Isolated yield. Values in parentheses were determined by GC internal standard method. ^d^ Electrolysis time 11 h. ^e^ Yields were calculated based on the GC conversion and selectivity.
